# Changes in facial appearance alter one’s sensitivity not only to the self but also to the outside world

**DOI:** 10.3389/fpsyg.2024.1426820

**Published:** 2024-09-10

**Authors:** Motoyasu Honma, Sayaka Yoshiba, Saya Miyamoto, Nanae Himi, Shugo Haga, Sumire Ogura, Koutaro Maki, Yuri Masaoka, Masahiko Izumizaki, Tatsuo Shirota

**Affiliations:** ^1^Department of Physiology, Showa University School of Medicine, Tokyo, Japan; ^2^Department of Oral Surgery, Tokyo Women’s Medical University Adachi Medical Center, Tokyo, Japan; ^3^Department of Oral and Maxillofacial Surgery, Showa University School of Dentistry, Tokyo, Japan; ^4^Department of Orthodontics, Showa University School of Dentistry, Tokyo, Japan

**Keywords:** facial appearance, jaw deformity, orthognathic surgery, self-evaluation, external evaluation

## Abstract

**Introduction:**

Changes in facial appearance due to orthognathic surgery are known to improve a patient’s postoperative quality of life, however, potential changes in cognitive function are unknown. This study examined the effects of changes in facial appearance due to orthognathic surgery on the sensitivity to self and to outside objects in patients with jaw deformities.

**Methods:**

Patients with jaw deformities (*n* = 22) and healthy controls (*n* = 30) were tested at 3 months preoperatively, at 1 month preoperatively, and at 1 month postoperatively to assess their impression of objects (positive, negative, and neutral pictures) and their evaluation of their own face and body.

**Results:**

The results showed that changes in facial appearance improved self-evaluation and increased their sensitivity to emotional objects even when the objects were identical. Furthermore, the improving rating for own face was associated with the sensitivity for objects.

**Discussion:**

The changes in facial appearance in patients may have helped to clear the sensitivity to these emotional objects. These findings may provide a new indicator of efficacy in orthognathic surgery.

## Introduction

The goals of treatments for jaw deformities include objective results such as restoring oral function and improving facial morphological disharmony, as well as subjective results such as eliminating psychological disorders (Yao et al., 2014) and improving social adaptability (Khechoyan, 2013; Takatsuji et al., 2015; Zamboni et al., 2019). The health-related quality of life (QOL), including physical state, psychological state, social state, economic state, and spiritual state, is reduced in patients with jaw deformities and body dysmorphic disorder (BDD), however, improvements in facial appearance achieved by orthognathic surgery have been reported to improve the QOL and mental health in postoperative patients (Finlay et al., 1995; Kim et al., 2009; IsHak et al., 2012; Bensoussan et al., 2014; Sun et al., 2018; Tachiki et al., 2018; Al-Hadi et al., 2019; Brucoli et al., 2019). It has been suggested that those patients are prone to self-recognition distortions due to complexes about their physical appearance (American Psychiatric Association, 2013), and that improvements in their physical appearance may have improved their self-recognition and thus their QOL. However, the QOL assessment addresses broad health and life-related items and does not assess specific cognitive functions. In this study, we asked whether when a patient’s facial appearance is improved, not only the recognition of the self, but also the recognition of the outside world is altered.

In order to examine changes in recognition of the outside world triggered by improving the physical facial appearance, this study focused on patients with jaw deformities who presented with facial deformities due to abnormalities in jaw size and shape. Jaw deformity is a disease showing an abnormal maxillofacial morphology, occlusal abnormalities, and aesthetic disharmony due to abnormalities in the morphology and position of the maxilla and mandible, and is thought to have a significant impact on the patient’s mental state (Yao et al., 2014; de Paula et al., 2019). Although there are many research reports on the surgical outcomes of orthognathic surgery, including surgical techniques and follow-up measures (Avinoam and Shetye, 2021; Park et al., 2015; Carbullido et al., 2022), internal evaluations have been limited to assessments of the QOL (Bensoussan et al., 2014; Tachiki et al., 2018). In other words, no studies have been reported that quantitatively investigated patients’ sensitivity change with improvement in facial appearance. Here we studied the impact of physical changes in the facial appearance of patients on their sensitivity to self and outside objects, and we assessed the relationship between the sensitivity to self/outside objects and the mental state based on a relationship of anxiety with the self-face evaluation (Pujol et al., 2013; Kim et al., 2016).

## Methods

### Participants

**Figure 1:**
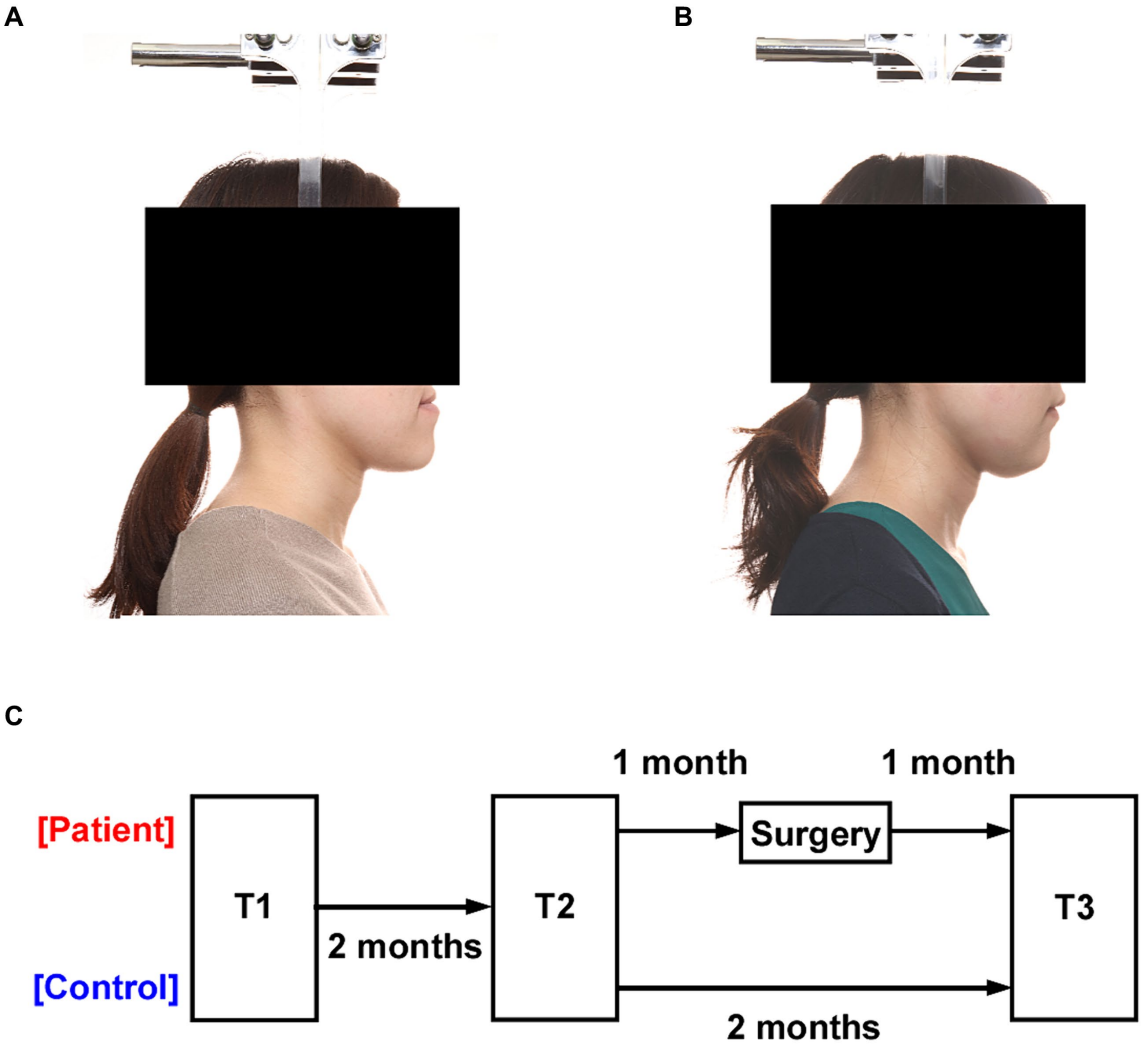
Experimental design. **(A)** Profile photograph of a patient with a jaw deformity. **(B)** Profile photograph of the patient after orthognathic surgery. **(C)** All participants were tested three times at two-month intervals (T1, T2, and T3). The interval between T1 and T2 was two months in both the patient and the healthy control groups, and T2 and T3 were performed one month before and one month after surgery in the patient group.

This study was conducted during the implementation of measures to prevent the spread of infectious diseases, and although 30 patients were expected to attend, only 22 attended. Participants in this study were 22 patients who required orthognathic surgery including bilateral sagittal split osteotomy (BSSRO) with or without Le Fort I osteotomy at our University Hospital between September 2020 and June 2021 (mean age: 24.3 years [range: 18–39], 14 females) (Figures 1A,B). Patients undergoing other surgical procedures were excluded. Twenty-two patients who met the exclusion criteria (age, comorbidities, and different surgical techniques) were included. A group of 30 healthy age—and sex-matched participants was recruited (mean age 23.5 years [range: 20–35], 20 females). The unpaired *t* test showed no difference between age (*p* = 0.966), and the chi-square test showed no difference among sex (*p* = 0.820). None of the participants had any previous psychiatric or neurological history.

### Surgery

All patients were treated according to standard orthodontic surgical procedures using multi-bracket appliances for preoperative and postoperative orthodontic treatment (Iijima et al., 2017). All orthognathic procedures were performed by oral surgeons at the Showa University Dental Hospital using either a single mandibular osteotomy (BSSRO: 1 jaw) (Le Fort I osteotomy and BSSRO: 2 jaws) (Dal Pont, 1961; Obwegeser, 1964) or simultaneous maxillary and mandibular osteotomies (Bell, 1975; Bell et al., 1975). After surgery, each patient was in an intermaxillary fixation for 5 days and required approximately 10 days of hospitalization.

### Procedures

Sensitivity evaluations of pictures (emotional intensity and preference), sensitivity ratings for face and body of each patient, and anxiety were assessed at 3 months preoperatively (T1), 1 month preoperatively (T2), and 1 month postoperatively (T3) (Figure 1C). The physician confirmed that the facial swelling in the patients had gone down before performing the T3. Thirty images (10 each with positive or negative emotional components and 10 neutral images; Supplementary Figure S1) were selected from the EmoMadrid emotional image database (Carretié et al., 2019). In the database, a valence and arousal of each of these pictures were assessed by an average sample of 146 volunteers per session, who evaluated an average of 155 pictures each. Sensitivity ratings included intensity of emotion (how much one feels emotion towards the images: 0–100) and preference (how much one ‘likes’ or ‘dislikes’ the image: 0–100) for the images using a visual analog scale (VAS). Preference ratings of one’s own face and body were also made using a VAS. The State–Trait Anxiety Inventory (STAI) was used to assess state and trait anxiety (Johnston, 1980) The STAI is a self-administered questionnaire with 20 questions each on trait anxiety and state anxiety using a 4-point scale. Trait anxiety reflects a personality trait that tends to cause anxiety. State anxiety reflects a temporary anxious reaction to a particular time, scene, event or object. The STAI has also been validated for use with Japanese people (Suzuki et al., 2000).

### Statistical analysis

Because the same sample was tested repeatedly in this experiment, we applied repeated measurement analysis. Repeated measures analysis of variance (RM-ANOVA) and multiple comparison test with Bonferroni correction were performed for each index (emotional and preference evaluations in each neutral, positive, and negative for objects; evaluations of face and body of self; state—and trait-anxiety). Test (T1, T2, and T3) and group (patients and healthy controls) were independent factors. The difference between T2 and T3 scores was defined as a degree of improvement. Additional analysis was also carried out by analysis of covariance (ANCOVA) with age as a covariate. Pearson’s correlation analysis was performed to test relationships in the degree of improvement between the indices. All tests were two-tailed. The results are presented as mean ± standard error of the mean and effect sizes (*η*^2^). Statistical significance was set at a *p* value <0.05. SPSS version 26 for Windows (IBM, Inc., Chicago, IL) was used for statistical analyses.

## Results

Data from the T1 in 2 of the 22 patients could not be checked due to technical problems and were therefore treated as missing values. To examine the effects of repetition for the same objects and time course (Figure 1C), one-way ANOVA was performed for the difference between T1 and T2 scores in both the patient and the healthy control groups. There was no significant difference in any of the indexes (Supplementary Table S1), which suggests that the effects of repetition and time course were insignificant.

### Ratings for each patient’s face increased after surgery

**Figure 2:**
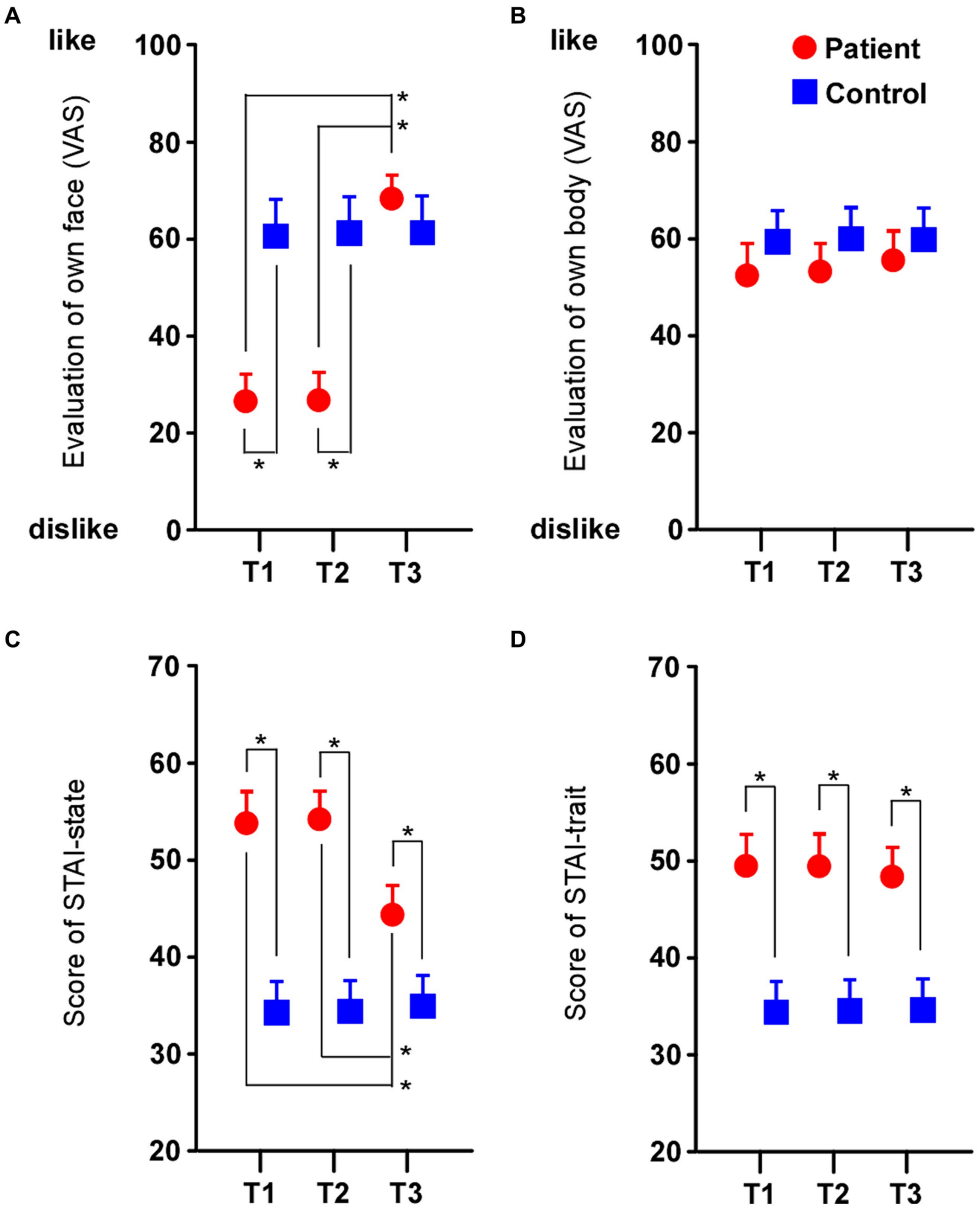
Rating of each subject’s face and body and assessment of anxiety. **(A)** Preference rating for each patient’s face was negative compared to the healthy controls before surgery (T1 and T2), and the rating became positive after surgery (T3). **(B)** The rating for each patient’s body was not changed before or after surgery in the patients or in the healthy controls. **(C)** The assessment of STAI-state in patients was low before surgery (T1 and T2) and improved after surgery (T3). **(D)** The assessment of STAI-trait in patients remained low after surgery compared to healthy controls (T3). Asterisks indicate significant differences (*p < 0.05). Error bars show the standard error of the mean.

In ratings of their own facial appearance (Figure 2A), the main effects of the test (F_2,96_ = 135.525, *p* < 0.0001, *η*^2^ = 0.738), group (F_2,48_ = 65.591, *p* < 0.0001, *η*^2^ = 0.577) and their interaction (F_2,96_ = 130.545, *p* < 0.0001, *η*^2^ = 0.731) were significantly different. Multiple comparison tests revealed that the preference rating in patients was negative compared to healthy controls in both T1 and T2 (respectively *p* < 0.0001), while in T3, the preference rating in patients increased towards the positive compared to T2 (*p* < 0.0001), and there was no significant difference between the groups (*p* = 0.398). On the other hand, in the rating of their own body appearance (Figure 2B), the main effects of the test (F_2,96_ = 1.203, *p* = 0.305, *η*^2^ = 0.024), group (F_2,48_ = 3.779, *p* = 0.058, *η*^2^ = 0.073) and their interaction (F_2,96_ = 0.462, *p* = 0.631, *η*^2^ = 0.010) were not significantly different. Thus, the rating for self-face changed after surgery, while the rating for self-body did not change after surgery. In addition, ANCOVA with age as a covariate showed that the results were similar to the case of non-covariate in own face (test: F_2,96_ = 13.187, *p* < 0.05, *η*^2^ = 0.219; group: F_2,48_ = 73.582, *p* < 0.0001, *η*^2^ = 0.610; interaction: F_2,96_ = 179.200, *p* < 0.0001, *η*^2^ = 0.792) and own body (test: F_2,96_ = 22.988, *p* = 0.055, *η*^2^ = 0.060; group: F_2,48_ = 3.729, *p* = 0.060, *η*^2^ = 0.074; interaction: F_2,96_ = 0.857, *p* = 0.428, *η*^2^ = 0.018).

### Assessment of anxiety improved after surgery

In the assessment of STAI-state (Figure 2C), the main effects of the test (F_2,96_ = 17.850, *p* < 0.0001, *η*^2^ = 0.271), group (F_2,48_ = 66.259, *p* < 0.0001, *η*^2^ = 0.580) and their interaction (F_2,96_ = 26.406, *p* < 0.0001, *η*^2^ = 0.355) were significantly different. Multiple comparison tests revealed that the assessment of STAI-state in patients was high compared to healthy controls in both T1 and T2 (respectively *p* < 0.0001), while the assessment of STAI-state in patients in T3 decreased significantly compared to T2 (*p* < 0.0001). On the other hand, in the assessment of STAI-trait (Figure 2D), although the main effect of group was significantly different (F_2,48_ = 52.896, *p* < 0.0001, *η*^2^ = 0.524), the test (F_2,96_ = 1.067, *p* = 0.348, *η*^2^ = 0.022) and their interaction (F_2,96_ = 0.120, *p* = 0.887, *η*^2^ = 0.003) were not significantly different. Thus, the assessment of STAI-state improved after surgery, while the STAI-trait did not change throughout all tests in the patients. In addition, ANCOVA with age as a covariate showed that the results were similar to the case of non-covariate in STAI-state (test: F_2,96_ = 3.616, *p* < 0.05, *η*^2^ = 0.071; group: F_2,48_ = 65.164, *p* < 0.0001, *η*^2^ = 0.581; interaction: F_2,96_ = 26.981, *p* < 0.0001, *η*^2^ = 0.364) and STAI-trait (test: F_2,96_ = 5.886, *p* < 0.005, *η*^2^ = 0.111; group: F_2,48_ = 51.892, *p* < 0.0001, *η*^2^ = 0.525; interaction: F_2,96_ = 0.169, *p* = 0.845, *η*^2^ = 0.004).

### Emotional sensitivity to objects increased after surgery

**Figure 3:**
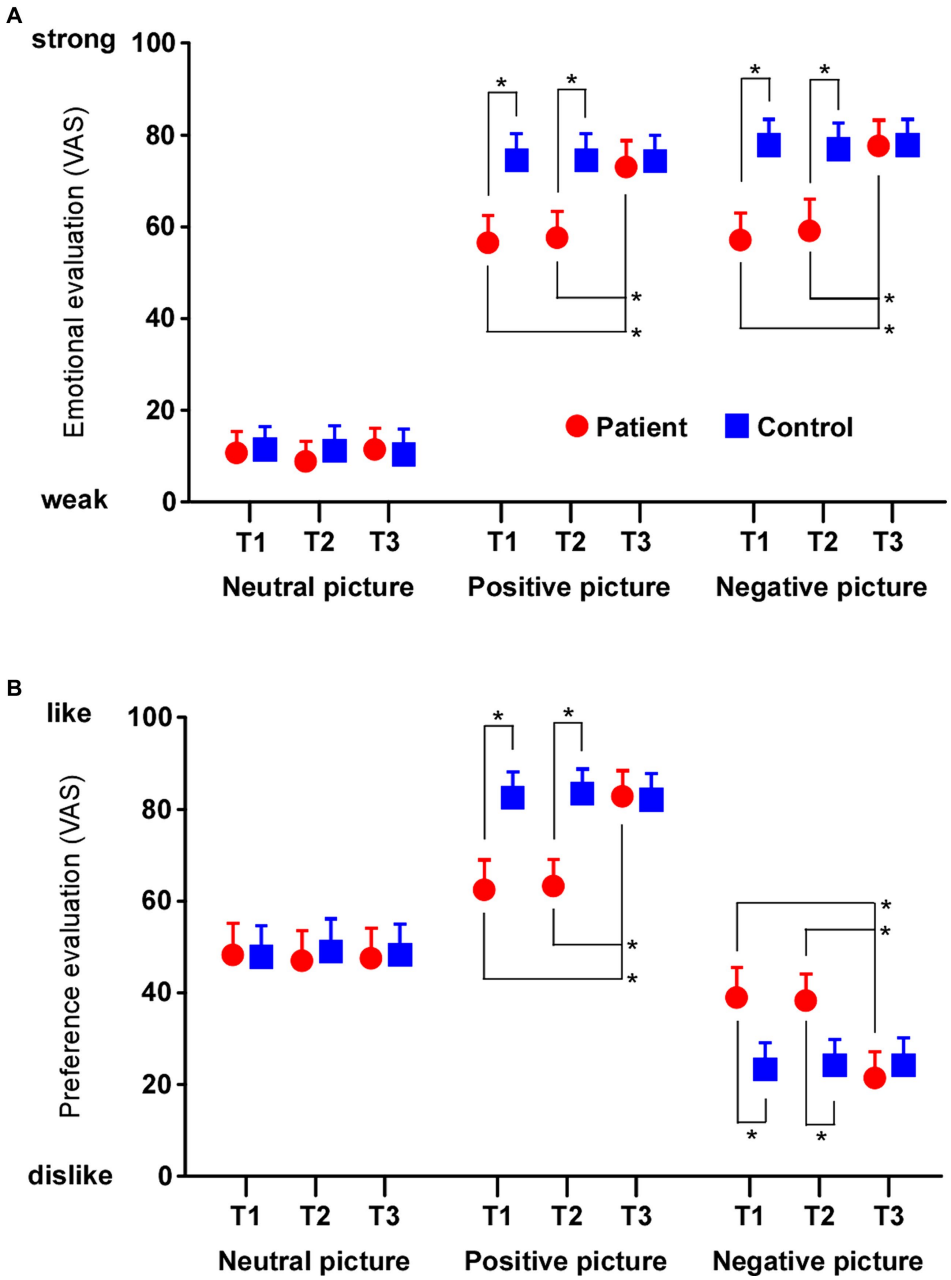
Changes in sensitivity after orthognathic surgery. **(A)** Emotional ratings for positive and negative objects became more sensitive after orthognathic surgery (T3) compared to before surgery (T1 and T2) in the patients. **(B)** Preference ratings for objects increased after orthognathic surgery. Patients, in the rating for negative objects, became more positive for positive objects and more negative for negative objects after surgery (T3). Asterisks indicate significant differences (*p < 0.05). Error bars indicate the standard error of the mean.

In the emotional rating for neutral objects (Figure 3A), the main effects of the test (F_2,96_ = 0.091, *p* = 0.913, *η*^2^ = 0.002), group (F_2,48_ = 43.447, *p* < 0.0001, *η*^2^ = 0.475) and their interaction (F_2,96_ = 0.310, *p* = 0.731, *η*^2^ = 0.006) were significantly different. In the rating for positive objects, the main effects of the test (F_2,96_ = 55.919, *p* < 0.0001, *η*^2^ = 0.538), group (F_2,48_ = 43.447, *p* < 0.0001, *η*^2^ = 0.475) and their interaction (F_2,96_ = 55.707, *p* < 0.0001, *η*^2^ = 0.537) were significantly different. Multiple comparison tests revealed that the patients, in the rating for positive objects, were rated as weak compared to healthy controls in both T1 and T2 (respectively *p* < 0.0001). Furthermore, the patients in T3 were rated as strong compared to T2 in the rating for positive objects (*p* < 0.0001) and there was no significant difference between patients and healthy controls in T3 (*p* = 0.531). In the rating for negative objects, the main effects of the test (F_2,96_ = 77.836, *p* < 0.0001, *η*^2^ = 0.619), group (F_2,48_ = 40.593, *p* < 0.0001, *η*^2^ = 0.458) and their interaction (F_2,96_ = 66.374, *p* < 0.0001, *η*^2^ = 0.580) were significantly different. Multiple comparison tests revealed that the patients, in the rating for negative objects, were rated as weak compared to healthy controls in both T1 and T2 (respectively *p* < 0.0001). Furthermore, the patients in T3 were rated as strong compared to T2 in the rating for negative objects (*p* < 0.0001) and there was no significant difference between patients and healthy controls in T3 (*p* = 0.997). In brief, the emotional sensitivity to emotional objects became strong after surgery, although the sensitivity to neutral objects did not change throughout all tests in the patients. In addition, ANCOVA with age as a covariate showed that the results were similar to the case of non-covariate in neutral (test: F_2,96_ = 1.985, *p* = 0.143, *η*^2^ = 0.041; group: F_2,48_ = 0.104, *p* = 0.748, *η*^2^ = 0.002; interaction: F_2,96_ = 0.390, *p* = 0.678, *η*^2^ = 0.008), positive (test: F_2,96_ = 10.659, *p* < 0.0001, *η*^2^ = 0.185; group: F_2,48_ = 42.277, *p* < 0.0001, *η*^2^ = 0.474; interaction: F_2,96_ = 58.430, *p* < 0.0001, *η*^2^ = 0.554), negative (test: F_2,96_ = 2.912, *p* = 0.059, *η*^2^ = 0.058; group: F_2,48_ = 36.847, *p* < 0.0001, *η*^2^ = 0.439; interaction: F_2,96_ = 39.656, *p* < 0.0001, *η*^2^ = 0.458).

### Preference for objects became clear after surgery

In the preference rating for neutral objects (Figure 3B), the main effects of the test (F_2,96_ = 2.896, *p* = 0.060, *η*^2^ = 0.057), group (F_2,48_ = 2.896, *p* = 0.060, *n* = 0.057) and their interaction (F_2,96_ = 2.896, *p* = 0.060, *η*^2^ = 0.057) were significantly different. In the rating for positive objects, the main effects of the test (F_2,96_ = 79.850, *p* < 0.0001, *η*^2^ = 0.625), group (F_2,48_ = 37.465, *p* < 0.0001, *η*^2^ = 0.438) and their interaction (F_2,96_ = 84.493, *p* < 0.0001, *η*^2^ = 0.638) were significantly different. Multiple comparison tests revealed that the patients, in the rating for positive objects, were rated as ‘dislike’ compared to healthy controls in both T1 and T2 (respectively *p* < 0.0001). Furthermore, the patients in T3 were rated as ‘like’ compared to T2 in the rating for positive objects (*p* < 0.0001) and there was no significant difference between patients and healthy controls in T3 (*p* = 0.947). In the rating for negative objects, the main effects of the test (F_2,96_ = 36.036, *p* < 0.0001, *η*^2^ = 0.429), group (F_2,48_ = 9.721, *p* = 0.003, *η*^2^ = 0.171), and their interaction (F_2,96_ = 42.833, *p* < 0.0001, *η*^2^ = 0.472) were significantly different. Multiple comparison tests revealed that the patients, in the rating for negative objects, were rated as ‘like’ compared to healthy controls in both T1 and T2 (respectively *p* < 0.0001). Furthermore, the patients in T3 were rated as ‘dislike’ compared to T2 in the rating for negative objects (*p* < 0.0001) and there was no significant difference between patients and healthy controls in T3 (*p* = 0.997). In brief, the preference for emotional objects became clearer about ‘likes’ and ‘dislikes’ after surgery, although the preference for neutral objects did not change throughout all tests in the patients. In addition, ANCOVA with age as a covariate showed that the results were similar to the case of non-covariate in neutral (test: F_2,96_ = 1.947, *p* = 0.148, *η*^2^ = 0.040; group: F_2,48_ = 0.198, *p* = 0.658, *η*^2^ = 0.004; interaction: F_2,96_ = 2.375, *p* = 0.099, *η*^2^ = 0.048), positive (test: F_2,96_ = 10.269, *p* < 0.0001, *η*^2^ = 0.179; group: F_2,48_ = 36.191, *p* < 0.0001, *η*^2^ = 0.435; interaction: F_2,96_ = 75.195, *p* < 0.0001, *η*^2^ = 0.615), negative (test: F_2,96_ = 1.143, *p* = 0.323, *η*^2^ = 0.024; group: F_2,48_ = 8.225, *p* < 0.01, *η*^2^ = 0.149; interaction: F_2,96_ = 34.932, *p* < 0.0001, *η*^2^ = 0.426).

### Improvement of rating for own face associated with the sensitivity for objects

**Figure 4:**
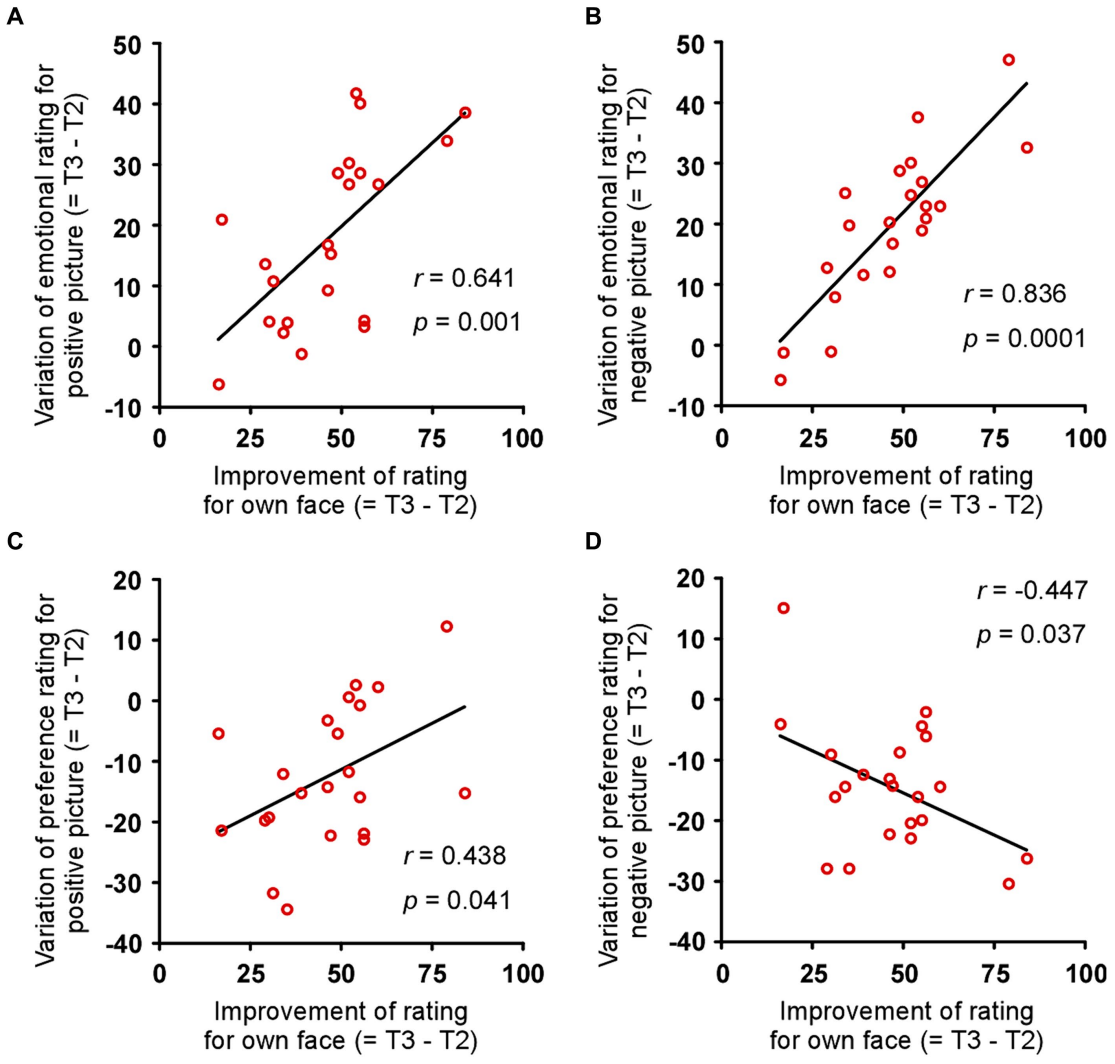
Association between improved rating of own face and improved sensitivity to objects. Changes in emotional rating for **(A)** positive and **(B)** negative objects correlated with the improvement in the rating for each patient’s face and the amount of variation due to surgery. Changes in preference rating for **(C)** positive and **(D)** negative objects correlated with the improvement in rating for each patient’s face and the amount of variation due to surgery. *r* and *p* indicate Pearson’s correlation coefficient and *p* value, respectively.

The difference between tests 2 and 3 for each index in the patient group was calculated as the amount of variation due to surgery (= T3 – T2). The improvement in rating for own face correlated with the variation in emotional rating for the positive objects (Figure 4A: *r* = 0.641, *p* = 0.001) and negative objects (Figure 4B: *r* = 0.836, *p* = 0.0001), but not the neutral objects (*r* = −0.098, *p* = 0.664). Similarly, the improvement in rating for own face correlated with the variation in preference rating for the positive objects (Figure 4C: *r* = 0.438, *p* = 0.041) and negative objects (Figure 4D: *r* = −0.447, *p* = 0.037) but not the neutral objects (*r* = 0.145, *p* = 0.519). On the other hand, the improvement in rating for own body did not correlate with the variation in all emotional and preference ratings (all *p* > 0.05). Furthermore, the improvements in STAI-state and -trait did not correlate with the variation in all emotional and preference ratings (all *p* > 0.05). These results show that, in the patients, the change in sensitivity to their own face is related to the change in sensitivity to emotional objects.

## Discussion

This study showed that an improvement in the physical appearance of the face leads to a positive evaluation of outside objects, as well as the evaluation of the self, and also to an improvement in anxiety tendencies. In particular, with regard to the evaluation of outside objects, the results showed that the intensity of evaluation approached those of healthy subjects after surgery. These results suggest that impressions of outside objects change postoperatively, even when the objects were identical.

The finding of a post-operative increase in patients’ assessment of their own facial appearance may reflect the patients’ satisfaction with the success of the operation and the fulfillment of their wishes. On the other hand, there was no change in their body image, which was used as a control indicator. This suggests that surgical changes in facial appearance do not necessarily change people’s assessment of their non-surgical body. In other words, it suggests that the facial surgery did not affect all ratings, but that the effect was limited to ratings of one’s own face. In terms of the patient’s evaluation of outside objects, post-operative scores on the emotion rating task were higher for both positive and negative objects compared to healthy subjects. On the other hand, scores in the preference rating task became more positive for positive objects and more negative for negative objects. However, neutral objects showed no change before or after surgery. These results suggest that the influence of the physical change in appearance is limited to the evaluation of the emotional component.

Emotions associated with anxiety are processed in the amygdala and affect several regions of the brain (Ghasemi et al., 2022; Ruggiero et al., 2021; Kami et al., 2022). In particular, the hippocampus is more likely to retain emotion-related memories (Mathis and Lecourtier, 2017; Durieux et al., 2020). Anxiety is also known to cause cognitive distortions and non-adaptive behaviour (Alladin and Amundson, 2016; Park et al., 2022). The change in patients’ anxiety in the present study may reflect how high the patients’ preoperative mental state was in terms of anxiety tendencies and how these tendencies improved postoperatively. However, the correlation analysis showed that the improvement in the rating of their own face was associated with the improvement in the rating of emotional objects, whereas the improvement in the STAI state was not associated with an improvement in the rating of emotional objects. The results of this intra-individual correlation suggest that it is not simply an improvement in anxiety that causes the improvement in outside object ratings, but that the improvement in self-face ratings causes changes in the improvement of outside object ratings.

As a possible brain mechanism, the orbitofrontal cortex (OFC) may be a key. A previous study reported that in healthy people, the larger the OFC, the more flexible they were to stress and the fewer anxiety symptoms they had (Moore et al., 2018). Patients with BDD have also been found to show increased activity in the left OFC and bilateral parts of the caudate nucleus when viewing their own face, and increased functional connectivity between the OFC and the amygdala when viewing emotional images (Feusner et al., 2010; Borgers et al., 2022). The exact role of the OFC is not clear, but one interpretation is that the OFC may over-regulate the amygdala in patients with BDD. The brain mechanisms in the patients in this study may be similar to those in BDD.

Previous studies of jaw deformities have typically employed the QOL indices (Finlay et al., 1995; Kim et al., 2009; IsHak et al., 2012; Bensoussan et al., 2014; Tachiki et al., 2018; Al-Hadi et al., 2019), which primarily assess physical and mental health in broad terms, and it has been difficult to examine a detailed internal state that mediates the relationship between “improvement in facial appearance” and “improvement in QOL” using QOL indices alone. The present study employed more specific emotional and anxiety indices to examine the internal state, thereby demonstrating that emotion and anxiety are involved in the internal state that mediates the relationship. The results indicated that orthognathic surgery alters “cognitive function” and “psychiatric disease tendency” in patients with jaw deformities.

## Limitations and directions for future research

The current study has several limitations. First, this study collected and analyzed behavioral data but did not measure brain imaging or neurotransmitters. Future studies should use functional magnetic resonance imaging to observe and investigate potential relationships between brain activity and sensitivity rating. In particular, the activity of the OFC, as well as the amygdala and hippocampus, may be significantly altered before and after surgery. Second, from the perspective of obsessive-compulsive disorder, abnormalities in the neurotransmitter serotonin may also be an important factor (Phillipou et al., 2016; Dong et al., 2019). It is also necessary to measure changes in the secretion of serotonin and other neurotransmitters. In addition to the jaw deformities assessed in this study, it is also necessary to characterize changes in healthy individuals who have undergone orthognathic surgery, paying attention to the association with psychiatric disorders. Third, while the physician confirmed that the facial swelling in the patients had gone down before performing the T3, there was little objectivity in the assessment of that swelling. Future studies must develop objective methods to assess swelling, and then it also required to conduct a long-term postoperative observation. Forth, the indicator on mental disorders was limited to anxiety in this study. Because that jaw deformities are facial disfigurement, it seems that patients with jaw deformities experience strong complexes and excessive negative feelings about their appearance, and it is thought that the patients have characteristics of various mental disorders in addition to anxiety disorders, such as social anxiety disorder, sleep disorders, and dysmorphophobia. It is possible that these characteristics also form individual differences in cognitive function and mental disorder tendencies that are affected by orthognathic surgery. For this reason, it would be beneficial to gain a deeper understanding of the individual characteristics of patients in many mental disorder-related assessments, and then it would be valuable to conduct further experiments to gain a deeper insight into these characteristics. Fifth, the study was conducted in a case–control design, with patient and healthy groups being compared. The ideal approach would have been to divide the jaw deformity patients into two groups, one with surgery and one without, and to conduct the experiment in a random control design. However, it was difficult to extend the patients’ surgery dates significantly. The use of a random control is crucial to minimize confounding factors, given the possibility that other psychiatric disorders, in addition to anxiety, may exert an influence. Finally, this study had a small sample size, so it is difficult to draw firm conclusions about the impact of orthognathic surgery on patients’ emotions and anxiety. Therefore, it is desirable to conduct experiments with a large sample size in future research. When doing so, it is necessary to consider the above-mentioned grasp of various mental disorder tendencies and a random control design. These findings could lead to the development of new intervention programs, such as improving the internal state after surgery by grasping the patient’s detailed internal state before surgery and managing it appropriately, and could make a significant contribution to the field of orthodontics.

## Conclusion

This study showed that facial orthognathic surgery alters not only the evaluation of the self but also the evaluation of the outside world. We did not find any studies that quantified changes in cognitive function, including evaluation of the outside world, as a result of changes in facial appearance, and these results might provide a new indicator of efficacy in orthognathic surgery. In particular, the results of an evaluation of sensitivity to the outside world suggest that patients had a blurred evaluation of objects with an emotional component before surgery, and that orthognathic surgery may have sharpened their recognition of those emotional objects. This may also indirectly be one factor that contributes to an improved QOL. Finally, the findings of this study do not constitute a recommendation for easy orthognathic surgery. However, if the assessment of cognitive function presented in this study is used as one of the main measures in research and clinical practice of disability related to appearance, it could provide more information and a clearer understanding regarding the illnesses and disorders that afflict appearance.

## Data availability statement

The raw data supporting the conclusions of this article will be made available by the authors, without undue reservation.

## Ethics statement

The studies involving humans were approved by Ethics Committee of Showa University. The studies were conducted in accordance with the local legislation and institutional requirements. The participants provided their written informed consent to participate in this study. Written informed consent was obtained from the individual(s) for the publication of any potentially identifiable images or data included in this article.

## Author contributions

MH: Conceptualization, Formal analysis, Funding acquisition, Methodology, Visualization, Writing – original draft, Writing – review & editing. SY: Data curation, Investigation, Methodology, Writing – review & editing. SM: Data curation, Writing – review & editing. NH: Data curation, Writing – review & editing. SH: Data curation, Writing – review & editing. SO: Data curation, Writing – review & editing. KM: Data curation, Writing – review & editing. YM: Formal analysis, Writing – review & editing. MI: Formal analysis, Writing – review & editing. TS: Supervision, Writing – review & editing.
