# Supplementary material for: Changes in facial appearance alter one’s sensitivity not only to the self but also to the outside world
Source: Front Psychol. 2024 Sep 10;15:1426820. doi: 10.3389/fpsyg.2024.1426820 (PMC11420145; doi:10.3389/fpsyg.2024.1426820)
Supplement: Supplementary file 1 [file Data_Sheet_1.PDF]

# Supplementary Information

**Changes in facial appearance alter one's sensitivity not only to the self but also to the outside world**

Motoyasu Honma, Sayaka Yoshiba, Saya Miyamoto, Nanae Himi, Shugo Haga, Sumire Ogura, Koutaro Maki, Yuri Masaoka, Masahiko Izumizaki, Tatsuo Shirota

Supplemental Table 1

Supplementary Figure 1

**Supplemental Table 1. Statistical results of differences between T1 and T2.**

| | F | *p* |
|---|---|---|
| Patients | | |
| Own face | 0.567 | 0.577 |
| Own body | 0.490 | 0.629 |
| STAI-state | 1.439 | 0.166 |
| STAI-trait | 1.157 | 0.262 |
| Emotional evaluation | | |
| Neutral | 0.409 | 0.687 |
| Positive | 1.955 | 0.065 |
| Negative | 1.469 | 0.148 |
| Preference evaluation | | |
| Neutral | 1.771 | 0.093 |
| Positive | 0.205 | 0.840 |
| Negative | 0.381 | 0.707 |
| Controls | | |
| Own face | 0.723 | 0.475 |
| Own body | 1.469 | 0.153 |
| STAI-state | 1.278 | 0.211 |
| STAI-trait | 1.361 | 0.184 |
| Emotional evaluation | | |
| Neutral | 1.439 | 0.161 |
| Positive | 1.000 | 0.326 |
| Negative | 0.948 | 0.351 |
| Preference evaluation | | |
| Neutral | 1.393 | 0.174 |
| Positive | 1.316 | 0.199 |
| Negative | 0.646 | 0.523 |

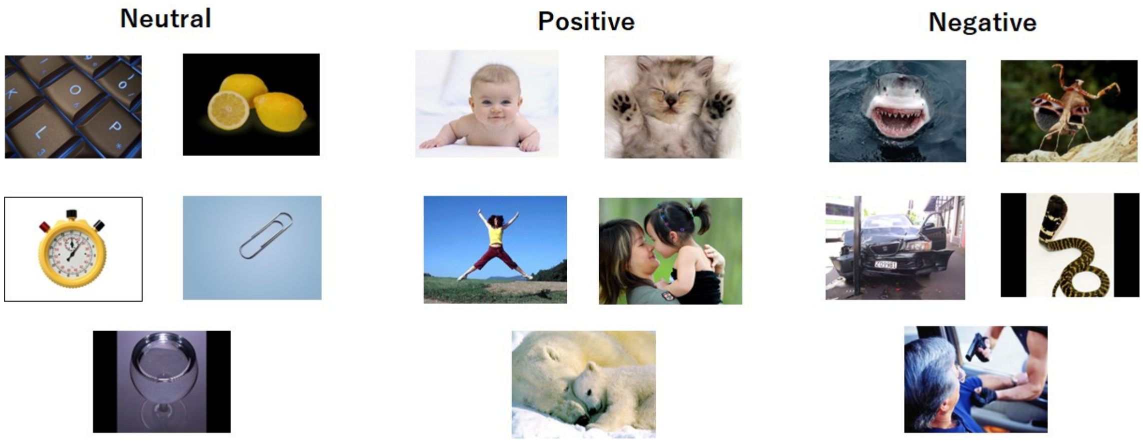


**Supplementary Figure 1.** A figure shows pictures used in the experiment. Those images were taken from the EmoMadrid emotional pictures database (Carretié et al., Motivation and Emotion, 2019, http://www.uam.es/CEACO/EmoMadrid).
